# Longitudinal analysis of antibody responses to *Plasmodium vivax* sporozoite antigens following natural infection

**DOI:** 10.1371/journal.pntd.0011907

**Published:** 2024-01-26

**Authors:** Pongsakorn Thawornpan, Justin Nicholas, Chayapat Malee, Piyawan Kochayoo, Kittikorn Wangriatisak, Pachara Tianpothong, Francis Babila Ntumngia, Samantha J. Barnes, John H. Adams, Patchanee Chootong

**Affiliations:** 1 Department of Clinical Microbiology and Applied Technology, Faculty of Medical Technology, Mahidol University, Bangkok, Thailand; 2 Center for Global Health and Interdisciplinary Research, College of Public Health, University of South Florida, Tampa, Florida, United States of America; 3 Department of Molecular Medicine, Morsani College of Medicine, University of South Florida, Tampa, Florida, United States of America; Academic Medical Center: Amsterdam UMC Locatie AMC, NETHERLANDS

## Abstract

**Background:**

*P*. *vivax* malaria is a major global health burden hindering social and economic development throughout many tropical and sub-tropical countries. Pre-erythrocytic (PE) vaccines emerge as an attractive approach for the control and elimination of malaria infection. Therefore, evaluating the magnitude, longevity and prevalence of naturally acquired IgG antibody responses against PE candidate antigens is useful for vaccine design.

**Methodology/Principal findings:**

The antigenicity of five recombinant PE antigens (PvCSP-VK210, PvSSP3, PvM2-MAEBL, PvCelTOS and PvSPECT1) was evaluated in plasma samples from individuals residing in low transmission areas in Thailand (Ranong and Chumphon Provinces). The samples were collected at the time of acute vivax malaria and 90, 270 and 360 days later. The prevalence, magnitude and longevity of total IgG and IgG subclasses were determined for each antigen using the longitudinal data. Our results showed that seropositivity of all tested PE antigens was detected during infection in at least some subjects; anti-PvCSP-VK210 and anti-PvCelTOS antibodies were the most frequent. Titers of these antibodies declined during the year of follow up, but notably seropositivity persisted. Among seropositive subjects at post-infection, high number of subjects possessed antibodies against PvCSP-VK210. Anti-PvSSP3 antibody responses had the longest half-life. IgG subclass profiling showed that the predominant subclasses were IgG1 and IgG3 (cytophilic antibodies), tending to remain detectable for at least 360 days after infection.

**Conclusions/Significance:**

The present study demonstrated the magnitude and longevity of serological responses to multiple PE antigens of *P*. *vivax* after natural infection. This knowledge could contribute to the design of an effective *P*. *vivax* vaccine.

## Author summary

The main objective of pre-erythrocytic (PE) vaccine development against *Plasmodium vivax* is to inhibit hepatocyte infection and the development of the hepatic parasite, thus limiting the subsequent invasion of red blood cells. Although immunization with irradiated sporozoites induces immune responses against the circumsporozoite protein (CSP), PE vaccines based on recombinant or synthetic CSP alone have not generated protective immunity. Investigating immune responses to sporozoite proteins which play a key role in PE development could help improve vaccine design strategies. Here, we assessed the prevalence, magnitude and longevity of antibody responses against several PE proteins in vivax malaria patients living in an area of low malaria transmission in Southern Thailand. This study provides evidence that antibody responses to surface, micronemal and traversal proteins of sporozoites developed during acute infection. Although the magnitude of IgG responses to all tested PE antigens declined with time, seropositivity to these antigens was maintained after infection. Future studies demonstrating functional activity of naturally-acquired antibodies against sporozoite invasion of hepatocytes would be invaluable to our understanding of anti-PE immunity and vaccine development.

## Introduction

*Plasmodium vivax* is one of the main agents responsible for malaria and high morbidity and mortality [1]. *P*. *vivax* has distinct biological characteristics, including a dormant hypnozoite stage in the liver that can cause relapse and the early production of gametocytes that promote further transmission [2]. Unfortunately, genetic study revealed that the prevalence of drug-resistant strains of *P*. *vivax* is increasing at an alarming rate, thereby making the control, elimination and eradication of malaria difficult [3]. This necessitates the development of a long-lasting and fully effective vaccine against *P*. *vivax* to accelerate its elimination [4,5].

Nowadays, pre-erythrocytic (PE) or anti-infective vaccines stand out as the most attractive approach for malaria prevention, by targeting parasites at the early phase of infection and reducing the severity of clinical symptoms. Experimental evidence in *P*. *falciparum* infections demonstrates that repeated exposure to high doses of chemically-, genetically- or radiation-attenuated sporozoites can induce protection [6–8]. However, a phase III clinical trial of the PE vaccine RTS,S/ASO1 showed limited efficacy and relatively short-lived protection [9–11]. To improve this efficacy, a second generation of *P*. *falciparum* CSP-based subunit vaccine was designed to promote the development of long-lived, affinity-matured plasma cells that maintain protective titers of anti-sporozoite antibody. In contrast to *P*. *falciparum*, very few *P*. *vivax* PE vaccines have progressed to clinical trials [12–14]. Therefore, a deeper understanding of the humoral responses against *P*. *vivax* PE antigens may help in the assessment of their potential as candidate vaccine antigens.

PE antigens have been selected as potential vaccine candidates based on their involvement in sporozoite motility and hepatocyte invasion, and being targets of neutralizing antibodies. These include Circumsporozoite protein (CSP) [15], Sporozoite surface protein 3 (SSP3) [16], Sporozoite protein essential for cell traversal 1 (SPECT1) [17,18] and Membrane-associated erythrocyte binding-like protein (MAEBL) [19]. CSP and SSP3 are surface-exposed sporozoite antigens and it is postulated that they become easily recognizable by immune antibodies, thereby blocking sporozoite invasion into erythrocytes. CSP is the dominant sporozoite surface protein and the long-time leading PE vaccine candidate [20]. Previous studies revealed that irradiated sporozoites induce CSP-specific immune responses which are associated with sterile immunity [21–23]. Even though *P*. *falciparum* circumsporozoite protein (PfCSP) is incorporated in the RTS,S vaccine and shows high efficacy in some settings [24], *P*. *vivax* CSP-based vaccines did not show satisfactory levels of protective efficacy in initial clinical trials [25–27]. SSP3 was found to be essential for sporozoite motility and infectivity [16,28]. Although there is no report associating naturally acquired immunity to SSP3 with protection, genetic analysis has shown that PbSSP3^-^ mutant sporozoites have decreased exoerythrocytic maturation [28]. The sporozoite micronemal (MAEBL) and traversal proteins (Cell-traversal protein for ookinetes and sporozoites; CelTOS and Sporozoite protein essential for cell traversal 1; SPECT1) are also promising PE vaccine candidates. MAEBL is a sporozoite attachment protein vital for mosquito salivary gland infection and hepatocyte invasion [19, 29–32]. Anti-sera targeting the ectodomain M2 of MAEBL demonstrated inhibition of PE development and lethal *P*. *yoelii* infection [29, 30]. CelTOS play a crucial role in cell-traversal ability [33]. A study of *P*. *berghei celtos* genetic disruption found that the absence of this gene reduced sporozoite infectivity in the liver and significantly inhibited cell traversal by ookinetes in mosquito midgut wall and sporozoite traversal of different kinds of cells [34]. SPECT1 is a PE immune target due to the key role it plays in the malaria parasite’s crossing the dermis and liver sinusoidal wall prior to invasion of hepatocytes [35–37]. Up to now, none of the PE vaccines tested achieved satisfactory rates of protection, and the induced responses tended to be short-lived or highly strain specific. As such, it is of great importance to further assess the magnitude and durability of antibodies against these PE antigens in *P*. *vivax*-infected subjects to determine whether they have sufficient immunogenicity to enhance and prolong the protective effects of PE vaccines.

In the present study, we sought to assess the prevalence, magnitude and durability of antibody responses against five different PE antigens: PvCSP-VK210, PvSSP3, PvSPECT1, PvM2-MAEBL and PvCelTOS. The half-life of each PE antigen-specific total IgG was estimated using exponential curve fitting models. Moreover, the magnitude and longevity of IgG subclass antibodies against antigens that showed the highest antigenicity (PvCSP-VK210 and PvSSP3) were determined. Details of antibody responses to PE antigens are beneficial to evaluate these antigens as candidate vaccines against vivax malaria.

## Methods

### Ethics statement

This study was approved by the Committee on Human Rights Related to Human Experimentation, Mahidol University Central Institutional Review Board (MU-IRB 2012/079.2408 and MU-CIRB 2021/281.2505). Written informed consent was obtained from each participant before blood collection. The criteria for recruitment of vivax malaria subjects were: (1) systolic blood pressure higher than 90 mmHg, (2) body temperature lower than 40°C, (3) hematocrit higher than 25%, and (4) age 18 years or above. Individuals who did not meet these criteria were excluded.

### Study site and participants

Heparinized blood samples were taken from subjects in malaria low-transmission areas in the southern part of Thailand (Chumphon and Ranong Provinces). Acute blood samples were collected at the Ministry of Public Health malaria clinics (Vector Borne Disease Units 11.4.2 and 11.5.3) and convalescent samples (day 90, 270 and 360) were collected at participants’ residences from August 2017 to December 2022. To assess naturally-acquired IgG responses to *P*. *vivax* PE antigens, a cross-sectional study was conducted that included *P*. *vivax*-infected subjects during at acute phase or day 0 (n = 104) and *P*. *vivax* subjects who recovered from infection for 90 days (n = 41), 270 days (n = 30) and 360 days (n = 27). The acute subjects who showed seropositive responses to PvCSP-VK210 (n = 35) or PvSSP3 (n = 28) were selected for assessment of IgG subclass responses. Of the 104 acute-phase subjects, 20 individuals were selected in cohort study for monitoring anti-PE antibody titers at four studied time points (days 0, 90, 270 and 360). Of these 20 subjects in 1-year cohort study, 10 and 8 subjects were determined to have detectable levels of anti-PvCSP-VK210 and -PvSSP3 IgG subclass antibodies at post-infection, respectively. The demographic information of recruited subjects is summarized in S1 Fig and S1 Table.

All *P*. *vivax* infections in study subjects were documented microscopically by both thin and thick Giemsa-stained blood smears, and confirmed by nested PCR [38]. The patients who had symptomatic and positive both *P*. *vivax* parasitemia and PCR were enrolled as acutely-infected patients (n = 104). The recovery *P*. *vivax* subjects at 90 (n = 41), 270 (n = 30) and 360 days (n = 27) after infection in cross-sectional study were tested for blood smear and PCR before blood collection. Histories of prior malaria infections in subjects were obtained from the records of malaria clinics. All participants were treated with a full course of Primaquine and Chloroquine. In cohort study, blood samples from *P*. *vivax*-infected subjects were collected and tested by nested PCR every three months to detect sub-patent malaria. Staff conducted weekly house-to-house visits to estimate the incidence of clinical malaria over the study period. Blood samples from malaria-naïve, healthy subjects (n = 52) who lived in a non-malaria endemic area (Bangkok, Thailand) were collected and used as healthy controls (HCs).

### Recombinant protein production

The gene sequences coding for PvCSP-VK210 (PVP01_0835600), PvSSP3 including amino acid residue 19–203 (PVX_123155), PvSPECT1 (PVP01_1212300), PvCelTOS (PVP01_1435400) and PvM2-MAEBL including residue 594–1011 (PVP01_0948400) were acquired from the PlasmoDB database [39] (S2 Table). The signal peptides of PvCSP, PvSSP3 and PvSPECT1, as well as the transmembrane domain for PvSSP3, were excluded in the expressed recombinant proteins. For PvCSP-VK210 subdomains, sequences encoding N-terminal (residues 26–97) and C-terminal (residues 263–340) regions were used for production of recombinant proteins. The modified codon-optimized coding sequences were cloned into a pET21a+ expression vector with a C-terminal 6xHis-tag (Genscript, USA). Recombinant antigens were expressed in One Shot BL21 Star (DE3) *E*. *coli* (Invitrogen, USA). Bacterial cells were cultured in Luria broth until reaching an OD_600_ of 0.6–0.8, then protein expression was induced with final concentration of 1 mM IPTG for 4 hours at 37°C. PvSPECT1 and PvCelTOS were purified from clarified bacterial lysate while PvSSP3 and PvM2-MAEBL were purified from inclusion bodies (IB) by centrifugation after bacterial cell lysis with two buffers: buffer 1 (50 mM Tris, 0.5 M NaCl, 0.2 mM EDTA, 3% sucrose and 1% TritonX-100) and buffer 2 (50 mM Tris, 0.5 M NaCl, 0.2 mM EDTA and 3M Urea). The inclusion bodies were solubilized by solubilizing buffer (20 mM phosphate buffer, 6 M guanidine hydrochloride. Solubilized proteins were purified by Ni-NTA affinity chromatography coupled with ÄKTA pure chromatography system (Cytiva). Purified IB proteins were then refolded by rapid dilution method. The resulting proteins were dialyzed against phosphate-buffered saline (PBS), aliquoted and stored at -80°C. Purity of all proteins was assessed by SDS-PAGE (S2 Fig).

### Detection of total IgG against PE antigens

Levels of total IgG antibodies specific to PE antigens (PvCSP-VK210, PvSSP3, PvM2-MAEBL, PvCelTOS and PvSPECT1) were determined by indirect ELISA as previously reported [40]. Briefly, recombinant PE antigens at 2 μg/mL were coated on 96-well plates. Plasma (1:200 dilution) was added to wells followed by HRP-conjugated goat anti-human IgG (KPL, USA). Signal was developed with TMB substrate and read at 450 nm. Cut-off values were calculated using mean OD plus 2 SD of HCs (n = 52). Seropositivity was defined as a reactivity index (RI) greater than 1.0. For each plate, the study plasma and control samples (negative and positive controls) were incubated in the wells. Negative control wells were plasma of HCs (n = 2); positive control wells were plasma of *P*. *vivax*-infected subjects (n = 2) who showed the highest RI values. For kinetic studies, plasma from the same subject at different times were incubated on the same plate. All the samples were assessed in duplicate and mean values were used for analyses. Tetanus toxoid (Merck Millipore, Germany) was used as control protein for the detection of total IgG in *P*. *vivax* subjects and HCs and for the estimation of anti-PE antibody half-lives.

### Analysis of IgG subclass responses to PvCSP-VK210 and PvSSP3

To identify the predominant IgG subclass responses to PvCSP-VK210 and PvSSP3, indirect ELISAs were used for detection of IgG1, IgG2, IgG3 and IgG4 antibodies specific to these two antigens. The 96-well plates were coated with 2 μg/ml recombinant antigen. Plasma samples were added at a dilution of 1:100 and incubated for 1 hour at RT. After washing, mouse anti-human antibodies at dilution 1/500 for IgG1 and IgG3, and 1/1000 for IgG2 and IgG4 (Invitrogen Corp, CA, USA) were added and incubated for 1 hour at RT. Then, horseradish peroxidase (HRP)-conjugated goat anti-mouse IgG (Biolegend, USA) at 1/500 for IgG1 and IgG3, and at 1/1,000 for IgG2 and IgG4, were used for detection. The OD of the reaction was measured at 450 nm after the addition of the tetramethylbenzidine (TMB) enzyme substrate (Sigma Aldrich, USA). The positive controls were plasma (n = 2) from the *P*. *vivax*-infected patient who had the highest OD value for total IgG. A baseline OD was established using samples from HCs (n = 18). The RIs of IgG subclasses specific to PvCSP-VK210 and PvSSP3 were obtained by dividing the mean sample OD by the cut-off value. The cut-off values (mean OD + 2 SD) were calculated from a set of negative controls obtained from HC samples. A sample was defined as positive for a specific antibody if the RI was is equal to or greater than 1. All samples were assessed in duplicate and mean values were used for analyses.

### Data analysis

Statistical analyses were performed using GraphPad Prism version 8.4.3, (GraphPad Software, USA, https://www.graphpad.com). Statistical testing of serologic responses was performed using one-way ANOVA and Dunn’s multiple comparison tests. Statistical significance was considered as *p* < 0.05. Data analysis was performed using R version 4.1.1. To perform linear mixed effects regression, lme4 package was used and antibody responses were normalized as the percentage of maximal antigen-specific total IgG responses across all time points and plotted against days after maximal reactivity. Adjusted R^2^ (R^2^ adj) represents goodness of fit. Half-life was calculated by the following formula: In(0.5)/estimate. For correlation analysis, corrplot package version 0.90 was used for plotting and data was clustered by ward.D2 hierarchical agglomerative clustering method [41]. Heatmaps representing longitudinal serological responses and exponential curve fitting plots were made using ggplot2 package, version 3.3.5.

## Results

### Magnitude and seroprevalence of antibody responses to *P*. *vivax* PE antigens

**Fig 1:**
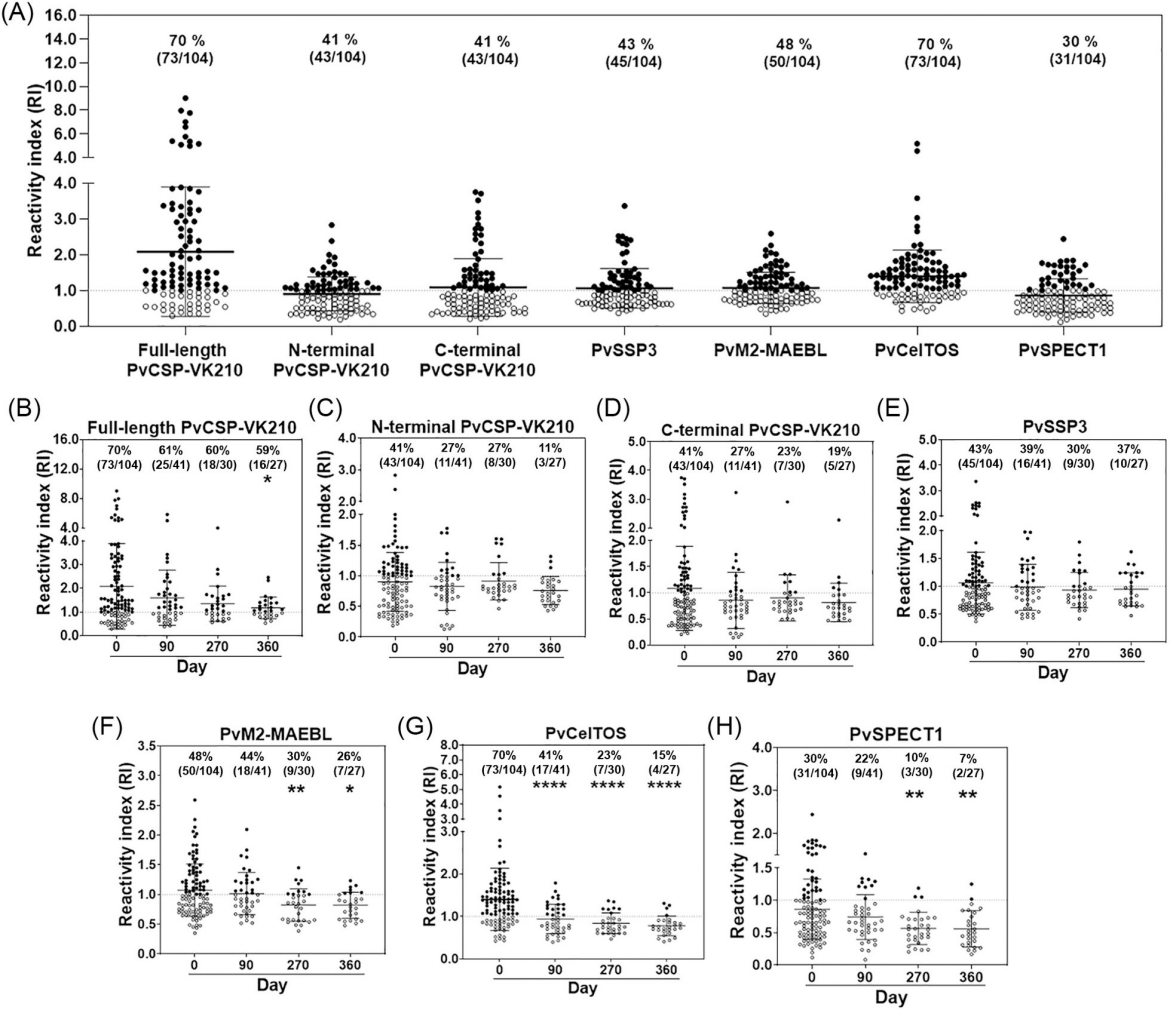
Antibody response and seroprevalence to *Plasmodium vivax* PE antigens. (A) Serological responses at acute phase expressed as reactivity index (RI) against 7 pre-erythrocytic stage antigens including full-length, N-terminal and C-terminal PvCSP-VK210, PvSSP3, PvM2-MAEBL, PvCelTOS, PvSPECT1. (B-H) Serological responses at 4 time points: at day 0 (n = 104), and recovery at day 90 (n = 41), 270 (n = 30) and 360 (n = 27). RI value was calculated by dividing mean OD value of each patient with cut-off value. Cut-off value was calculated from mean optical density (OD) values plus 2 SD of 52 HCs. The dashed line represents the cut-off value for seropositivity (RI = 1); samples were considered as seropositive if RI was greater than 1. Percentage shown on each scatter graph represents percentage of seropositive samples among total tested samples. Statistical testing was performed by one-way ANOVA: * p<0.05; ** p<0.005; *** p<0.0005; **** p<0.0001.

The magnitude and seroprevalence of antibodies against PE antigens were cross-sectionally assessed using plasma samples from *P*. *vivax*-infected patients at day 0 and after recovery on days 90, 270 and 360 (Fig 1). During acute phase, antibody responses (mean±SD) to full-length PvCSP-VK210 (2.08±1.80) were higher than to other PE antigens: N-terminal PvCSP-VK210 (0.90±0.48) and C-terminal PvCSP-VK210 (1.09±0.81), PvSSP3 (1.06±0.56), PvM2-MAEBL (1.07±0.44), PvCelTOS (1.40±0.73), PvSPECT1 (0.86±0.46). Seropositivities at acute phase were 70% (73/104) for full-length PvCSP-VK210, 41% (43/104) for both of N-terminal and C-terminal PvCSP-VK210, 43% (45/104) for PvSSP3, 48% (50/104) for PvM2-MAEBL, 70% (73/104) for PvCelTOS, 30% (31/104) for PvSPECT1. Of the 104 subjects, 8.7% (9/104) were positive to all PE antigens, while 55.8% (58/104) were positive to 3–6 of the PE antigens (Fig 1A).

During the recovery phase, a significant decrease was observed in antibody response to full-length CSP-VK210 at day 360 (1.17±0.45, p<0.05), while those to its N- and C-terminals showed no significant reduction (Fig 1B–1D). Interestingly, the levels of anti-SSP3 antibodies were stably maintained for up to 12 months post-infection (Fig 1E). Next, antibody responses to micronemal and traversal antigens were assessed (Fig 1F–1H). Antibody responses to PvM2-MAEBL (0.82±0.27) and PvSPECT1 (0.56±0.24) had decreased significantly (p<0.005 for both) by day 270 compared to those during acute malaria illness. Anti-PvCelTOS antibody titers were markedly reduced at day 90 (0.94±0.34, p<0.0001).

### Correlations among antibody responses specific to PE antigens

**Fig 2:**
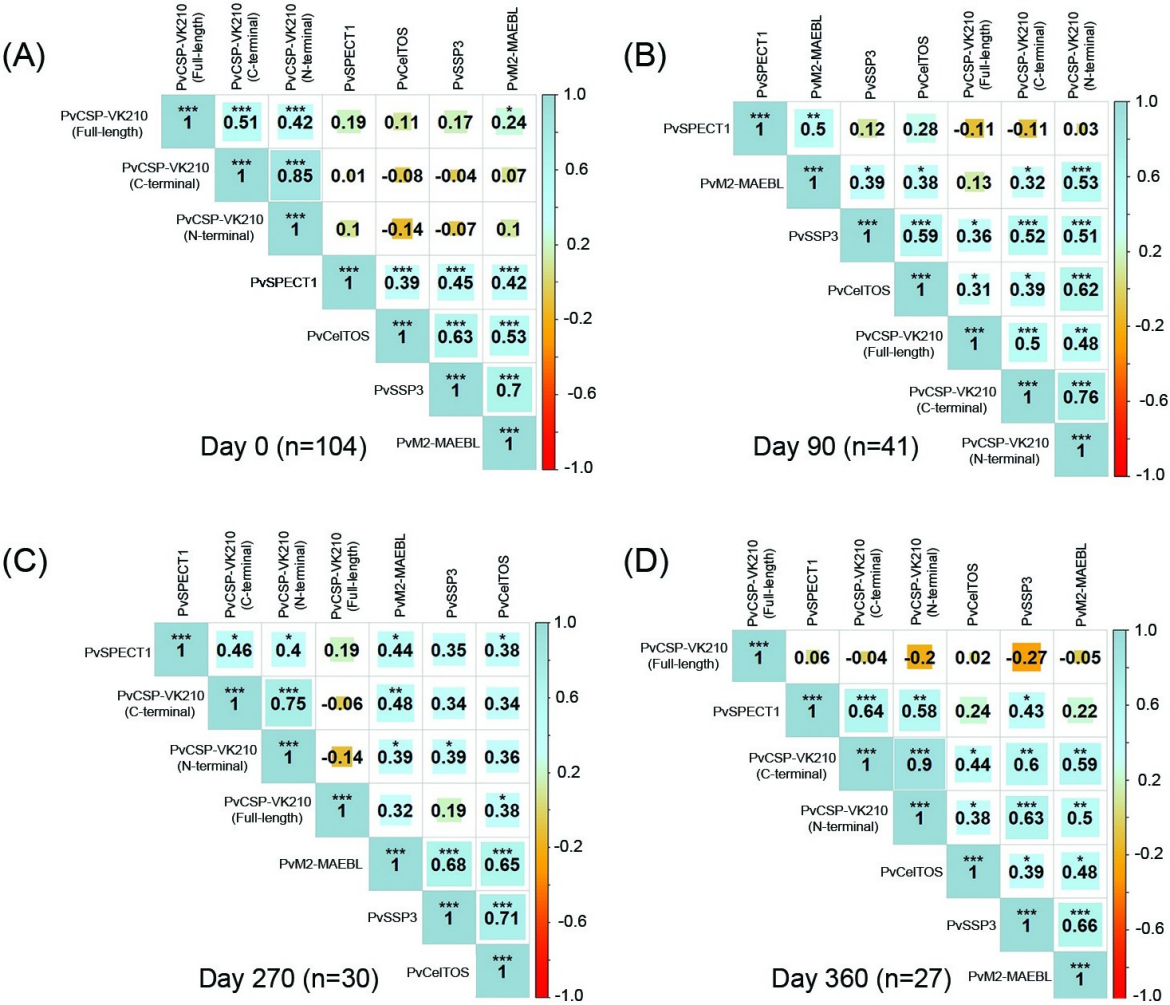
Correlation of antibody responses between specific PE antigens. (A-D) Antibody reactivity correlations between each pair of *P*. *vivax* PE antigens (full-length, N-terminal and C-terminal PvCSP-VK210, PvSSP3, PvM2-MAEBL, PvCelTOS and PvSPECT1). Pairwise Spearman’s correlations were determined between RI for each antigen at day 0 (acute phase), 90, 270, and 360. Correlation matrices were clustered using the ward.D2 hierarchical agglomerative clustering method. Shown are Spearman’s ρ and significance adjusted for multiple testing using the Holm method. * p<0.05; ** p<0.01; *** p<0.001. Spearman ρ>0.60 and adjusted p-value <0.01 are indicative of a strong positive and highly significant correlation. The coloring of the cells represents the sign of correlation: blue gradient for positive correlation and red gradient for negative correlation. The size of background squares represents degree of correlation corresponding to the values shown in each cell.

To identify correlations between antibody responses to different PE antigens, and at different timepoints (days 0, 90, 270 and 360), the responses were pairwise compared (Fig 2). A strong correlation was defined as a Spearman’s ρ greater than 0.6. At acute phase (day 0), PvSSP3 was positively and significantly correlated with PvM2-MAEBL (ρ = 0.7, *p* <0.001) and PvCelTOS (ρ = 0.63, *p* <0.001) (Fig 2A). At day 90, a significantly positive correlation was observed between N-terminal PvCSP-VK210 and PvCelTOS (ρ = 0.62, *p* <0.001) (Fig 2B). At day 270, three pairs of PE antigens were positively and significantly correlated: PvSSP3 and PvCelTOS (ρ = 0.71, *p* <0.001), PvM2-MAEBL and PvSSP3 (ρ = 0.68, *p* <0.001), as well as PvM2-MAEBL and PvCelTOS (ρ = 0.65, *p* <0.001) (Fig 2C). At day 360, significant and positive correlations were found between: PvSPECT1 and C-terminal PvCSP-VK210 (ρ = 0.64, *p* <0.001), C-terminal PvCSP-VK210 and PvSSP3 (ρ = 0.6, *p*<0.01), N-terminal PvCSP-VK210 and PvSSP3 (ρ = 0.63, *p*<0.001), and PvSSP3 and PvM2-MAEBL (ρ = 0.66, *p*<0.001) (Fig 2D). Strong positive and highly significant correlations were observed between C-terminal and N-terminal PvCSP-VK210 antigens at all timepoints: day 0 (ρ = 0.85, *p* <0.001), day 90 (ρ = 0.76, *p* <0.001), day 270 (ρ = 0.75, *p*<0.001) and day 360 (ρ = 0.9, *p*<0.001).

### Longitudinal seropositivity rates of anti-PE antibodies

**Fig 3:**
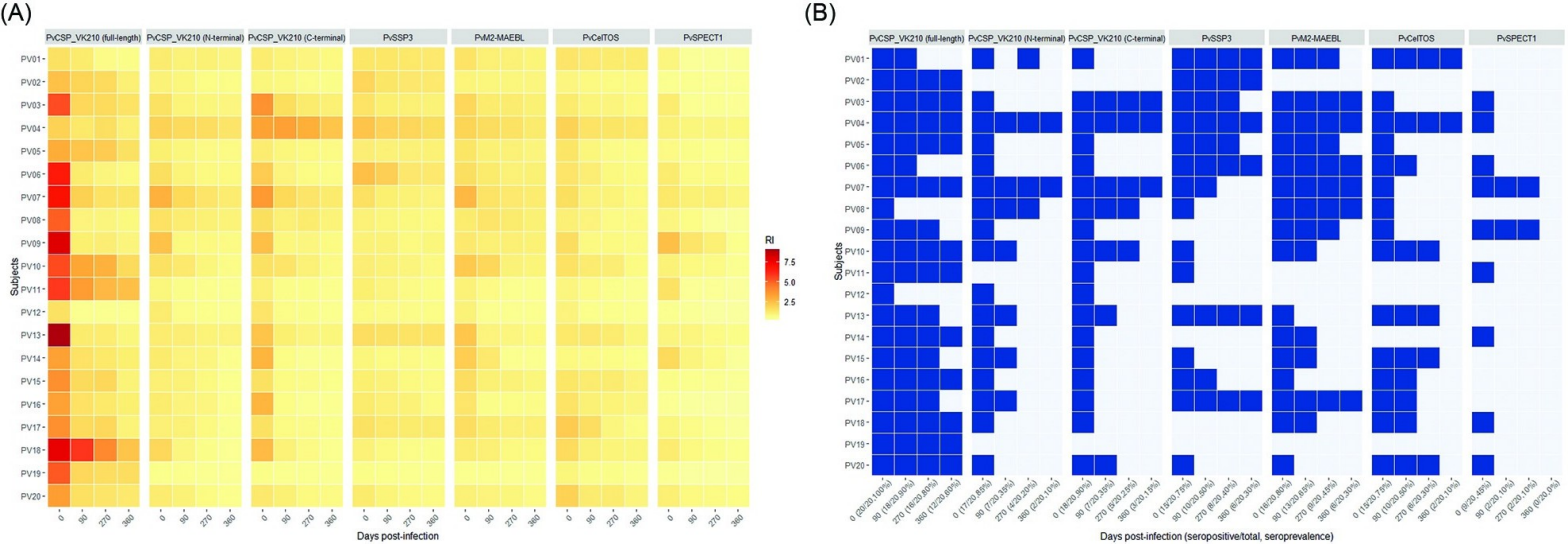
Longitudinal seropositivity rates against PE antigens of *P*. *vivax*. (A) Heatmap represents antibody responses against full-length, N-terminal and C-terminal PvCSP-VK210, PvSSP3, PvM2-MAEBL, PvCelTOS, and PvSPECT1 that were determined at four different timepoints (days 0, 90, 270 and 360) in individual *P*. *vivax* subjects (n = 20) residing in cohort study. (B) Seropositivity of IgG antibodies against PE antigens at different time points in *P*. *vivax* subjects (n = 20). Samples were considered as seropositive if the RI was greater than 1. Dark blue tiles represent seropositive samples at a given timepoint.

Seropositivity of anti-PE antibodies was determined after *P*. *vivax* infection in the cross-sectional survey. We then evaluated the longevity of seropositivity against PE antigens in twenty *P*. *vivax*-infected subjects whose plasma samples were available from each of the 4 timepoints (Fig 3A). Serological responses were considered long-lasting in subjects who remained seropositive for the whole 360 days. At acute phase (day 0), all subjects showed seropositive responses against full-length PvCSP-VK210, 85% for N-terminal PvCSP-VK210, and 90% for C-terminal PvCSP-VK210. After infection, seropositivity against PvCSP-VK210 decreased; 60% (12/20), 10% (2/20) and 15% (3/20) of subjects had persistent antibody responses to full-length, N terminal and C-terminal PvCSP-VK210 antigens through day 360, respectively (Fig 3B). Fifteen (75%) subjects were PvSSP3 seropositive at day 0, whereas only 6 (30%) subjects remained antibody positive at day 360. For micronemal antigens, 80%, 75% and 45% of *P*. *vivax*-infected-subjects produced antibody responses at day 0 to PvM2-MAEBL, PvCelTOS and PvSPECT1, respectively (Fig 3B). Persisting positivity was the antibody levels in 30% and 10% of subjects to PvM2-MAEBL and PvCelTOS at day 360, respectively. A great reduction of antibody to PvSPECT1 was seen post-infection, and all subjects were seronegative at day 360 (Fig 3B). In addition, there were 5 acutely infected subjects showed seropositive response to 5 PE antigens and two subdomains of PvCSP-VK210. These subjects did not remain seropositive to all tested PE antigens; only anti-PvCSP-VK210 antibodies displayed seropositivity at 1-year post-infection (Fig 3B).

### The anti-PE antibody half-lives

Since detectable anti-PE antibody was observed through day 360 in some *P*. *vivax*-infected subjects followed longitudinally, the half-lives of antibody responses were calculated. We employed a linear mixed-effect model using the reactivity index of each antigen as dependent variable, timepoint (days) as fixed effect, and subjects as random effect (Table 1). The analysis showed that SSP3 had the longest half-life (799 days, 95% CI = 686–961 days), while CSP-full length had the shortest (231 days, 95% CI = 191–291 days). The N-terminal and C-terminal PvCSP-VK210 antigens had half-lives of 443 days (95% CI = 372–521 days) and 323 days (95% CI = 267–410 days), respectively. Estimated half-lives of the other PE antigens were 517 days (95% CI = 422–666 days) for PvM2-MAEBL, 537 days (95% CI = 453–653 days) for PvCelTOS and 351 days (95% CI = 292–441 days) for PvSPECT1. For tetanus toxoid-specific antibody, half-life was estimated to be 1,397 days (95%CI = 1,047–2,107 days).

**Table 1:** Antibody half-life estimates using linear mixed-effects regression of antibody reactivity.

Antigen	Estimate	Std.error	Statistic	t1/2 (days)	Lower 95%CI_estimate	Higher 95%CI_estimate	Lower 95%CI	Higher 95%CI
PvCSP-VK210 (full-length)	-0.003	0.000309	-9.7	231	-0.00362	-0.00238	191	291
PvCSP-VK210 (N-terminal)	-0.0016	0.000133	-12	433	-0.00186	-0.00133	372	521
PvCSP-VK210 (C-terminal)	-0.00214	0.000225	-9.51	323	-0.00259	-0.00169	267	410
PvSSP3	-0.000867	0.0000728	-11.9	799	-0.00101	-0.000721	686	961
PvM2-MAEBL	-0.00134	0.000148	-9.04	517	-0.00164	-0.00104	422	666
PvCelTOS	-0.00129	0.000115	-11.2	537	-0.00153	-0.00106	453	653
PvSPECT1	-0.00197	0.000199	-9.9	351	-0.00237	-0.00157	292	441
Tetanus toxoid	-0.000496	0.0000833	-5.95	1397	-0.000662	-0.000329	1047	2107

**Fig 4:**
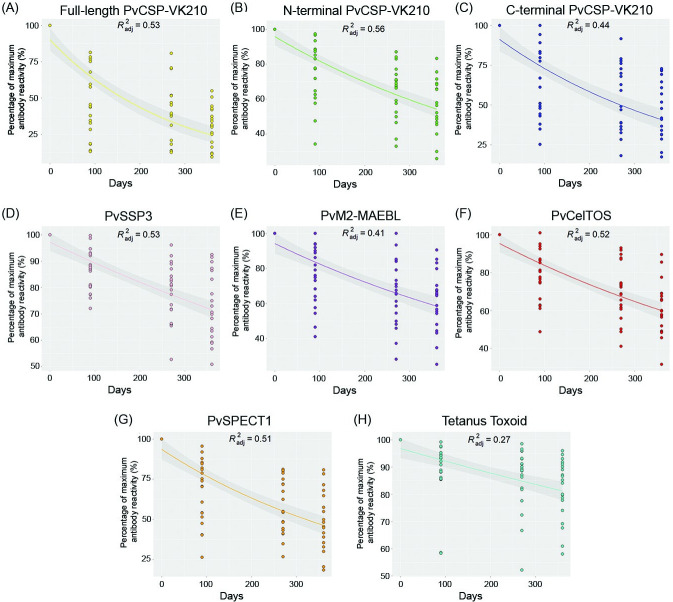
Half-life estimation of IgG antibodies against PE antigens using an exponential curve fitting model. (A-H) Half-lives of antibodies to PvCSP (full-length, N-terminal and C-terminal subdomains), PvSSP3, PvM2-MAEBL, PvCelTOS, PvSPECT1 and tetanus toxoid. For half-life estimation, antibody responses were normalized as the percentage of maximal antigen-specific total IgG responses across all timepoints and plotted against days after maximal reactivity. Adjusted R^2^ (R^2^ adj) represents goodness of fit.

Using an exponential curve fitting model, antibody responses were normalized as the percentage of maximal antigen-specific total IgG responses across four timepoints versus days after maximal seroreactivity (Fig 4). Based on adjusted *R*^2^ values (*R*^2^ *adj*), moderate goodness of fit (*R*^2^ *adj > 0*.*5*) was found in the antibody responses against N-terminal PvCSP-VK210 (*R*^2^ *adj* = 0.56), full-length PvCSP-VK210 and PvSSP3 (*R*^2^ *adj* = 0.53), PvCelTOS (*R*^2^ *adj* = 0.52), and PvSPECT1 (*R*^2^ *adj* = 0.51). Low goodness of fit was observed in the case of C-terminal PvCSP-VK210 (*R*^2^ *adj* = 0.44), PvM2-MAEBL (*R*^2^ *adj* = 0.41) and tetanus toxoid (*R*^2^ *adj* = 0.27).

### PE antigen-specific IgG subclass profiles

**Fig 5:**
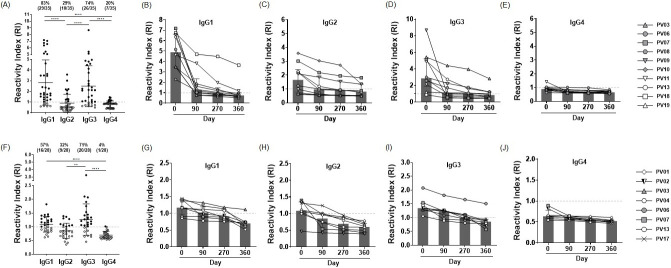
IgG subclass (IgG1-IgG4) responses against full-length PvCSP-VK210 and PvSSP3. (A) IgG subclass responses to PvCSP-VK210 at acute phase among *P*. *vivax*-infected subjects (n = 35). (B-E) Longitudinal IgG1-4 subclass responses to PvCSP-VK210 in *P*. *vivax*-infected subjects (n = 10) at days 0, 90, 270 and 360. (F) IgG subclass responses to PvSSP3 at acute phase among *P*. *vivax*-infected subjects (n = 28). (G-J) Longitudinal IgG1-4 subclass responses to PvSSP3 in *P*. *vivax* subjects (n = 8) at days 0, 90, 270 and 360. The reactivity index (RI) was calculated by dividing mean OD value of each patient with cut-off value. Cut-off value was calculated from mean optical density (OD) values plus 2SD of 18 HCs for full-length PvCSP-VK210 and 14 HCs for PvSSP3. Black dots represent seropositive samples while white dots represent seronegative. The dashed line represents the cut-off value for seropositivity (RI = 1); samples were considered as seropositive if RI was greater than 1. Statistical testing was performed by one-way ANOVA: * p<0.05; ** p<0.005; *** p<0.0005; **** p<0.0001.

Several studies have shown the association of IgG subclass responses and malaria protection [42–44]. Here, total IgG antibodies specific to full-length PvCSP-VK210 and PvSSP3 showed strong and durable responses over the tested timepoints. Thus, these two PE antigens were selected for detection of IgG subclass responses. For full-length PvCSP-VK210, the results showed that the mean (SD) of RI for IgG1, IgG2, IgG3 and IgG4 were 2.83 (2.11), 0.90 (0.82), 2.49 (1.91) and 0.80 (0.31), respectively. The response of IgG1 and IgG3 were significantly higher (p<0.0001) than those of IgG2 and IgG4. Seropositivities for IgG1, IgG2, IgG3 and IgG4 were 83% (29/35), 29% (10/35), 74% (26/35) and 20% (7/35), respectively (Fig 5A). Monitored kinetics of each IgG subclass in *P*. *vivax* subjects (n = 10) showed the median (IQR) of reactivity indices at days 0, 90, 270 and 360, respectively, were: 4.89 (3.04), 1.25 (0.78), 0.98 (0.47) and 0.74 (0.27) for IgG1; 1.66 (1.41), 0.97 (1.09), 0.90 (0.84) and 0.81 (0.70) for IgG2; 2.86 (2.86), 1.08 (1.37), 1.03 (0.81) and 0.84 (0.59) for IgG3; 0.89 (0.13), 0.71 (0.16), 0.67 (0.14) and 0.65 (0.14) for IgG4 (Fig 5B–5E).

For PvSSP3, antigen-specific IgG subclass profiles were determined in 28 *P*. *vivax*-infected subjects. The results showed that mean (SD) of RI for IgG1, IgG2, IgG3 and IgG4 was 1.09 (0.28), 0.86 (0.27), 1.27 (0.57), 0.70 (0.13), respectively. The IgG1 and IgG3 responses were significantly higher than those of IgG2 and IgG4 (p<0.005 and p<0.0001), respectively. Seropositivities for IgG1, IgG2, IgG3 and IgG4 were 57% (16/28), 32% (9/28), 71% (20/28) and 4% (1/28), respectively (Fig 5F). Monitored kinetics of each IgG subclass in *P*. *vivax*-subjects whose plasma were available at all four timepoints (n = 8) showed that the median (IQR) of reactivity indices at days 0, 90, 270 and 360 were: 1.17 (0.30), 1.03 (0.25), 0.91 (0.24) and 0.70 (0.10) for IgG1; 1.08 (0.29), 0.85 (0.31), 0.70 (0.34) and 0.60 (0.20) for IgG2; 1.34 (0.19), 1.23 (0.18), 1.01 (0.16) and 0.86 (0.14) for IgG3; 0.63 (0.09), 0.60 (0.06), 0.55 (0.05) and 0.52 (0.03) for IgG4, respectively (Fig 5G–5J). Taken together, IgG1 and IgG3 were predominant among the subclass responses to sporozoite surface protein antigens. Taken together, IgG1 and IgG3 were predominant among subclass responses to sporozoite surface protein antigens. Anti-PvCSP-VK210 IgG1 antibodies were persisted up to 360 days after infection, whereas IgG3 responses to this antigen were greatly declined since day 90 post-infection. The IgG1 response to PvSSP3 was seropositive for 90 days after infection in contrast to IgG3, which lasted for 270 days.

## Discussion

Insights into the serological responses against *P*. *vivax* PE antigens could provide the benefit in the development of anti-*Plasmodium vivax* vaccines [15,45,46]. Here, antibody responses against five PE candidates (PvCSP-VK210, PvSSP3, PvM2-MAEBL, PvCelTOS and PvSPECT1) and two subdomains of PvCSP-VK210 (N-terminal and C-terminal regions) were assessed in *P*. *vivax* patients during and after acute malaria infection. Our results showed the highest frequency of positive response during acute infection was to full-length PvCSP-VK210 (70%), compared to other PE antigens. We surveyed seroprevalence of anti-PvCSP-VK210 antibodies at recovery phase (Days 90, 270, 360) in comparison with acute phase of infection. At 360-day post-infection, anti-full-length PvCSP titers were significantly decreased, whereas antibody titers to N- and C-terminal did not greatly reduce. Of the 20 acute subjects in 1-year cohort study, 12 subjects without re-infection maintained positive IgG responses for at least one year. The calculated half-life of anti-PvCSP-VK210 antibodies was between 191 and 291 days. Similarly, high prevalence of PvCSP-VK210 seropositivity were also observed in regions of Brazilian Amazon and Western Thailand and high frequency of responders was consistent with the predominance of CSP-VK210 variants of *P*. *vivax* parasites in endemic areas [45–47]. A study by *Longley et*. *al*. showed a stable anti-PvCSP IgG titer for over a year in absence of detectable blood stage *P*. *vivax* infections [46]. Moreover, IgG responses to PvCSP-VK210 demonstrated that most of seropositive responses in symptomatic patients had previous *P*. *vivax* infection. Here, very low baseline of pre-existing anti-PvCSP antibodies in endemic villagers who lived in endemic areas with the absence of infection was detected and all samples showed seronegative responses with average RI (SD) of 0.63 (0.18) compared to 0.60 (0.20) of HCs. Altogether, our data support the development and persistence of IgG antibody responses to PvCSP following natural *P*. *vivax* infections.

Given the high immunogenicity of PvCSP, this antigen is potential to be included as a component in PE-based vaccine formulation. However, there are several gaps which must be addressed: (i) the prevalence of PvCSP allelic variants in endemic areas and its relationship with host immune responses as well as the contribution of pre-existing immune response to endemic strains in the variation of anti-PvCSP antibody responses; (ii) the inhibitory efficiency of anti-PvCSP antibody from high responders in inhibition of liver stage development. This might reflect the association between high antibody titers and malaria protection; (iii) given a higher frequency of antibody responses to full-length PvCSP compared to N- and C- termini, future studies should aim to identify target epitopes of naturally acquired anti-PvCSP inhibitory antibodies whether the predominant B epitopes are located at repeated region, as it was reported in *P*. *falciparum* CSP (PfCSP) [48]; (iv) since high antigenic diversity in C-terminal PfCSP displayed limited breadth of antibody response and evasion of cellular immunity [49], identification of T cell epitope on N- or C- subdomain of PvCSP and determination of the effect of these two subdomain polymorphisms on driving immune selection leading to strain-specific immunity are required; (v) the ability of PvCSP to induce development and persistence of long-lived memory B cell and plasma cell as well as its contribution to magnitude and durability of IgG responses. Our study in similar cohort of participants showed an expansion of atypical memory B cells during acute malaria and this MBC subset were persisted up to 3 months after parasite clearance [50], thus, it might account for the impairment of anti-PvCSP persistent responses. Together, a deeper understanding of immune responses to PvCSP could provide novel insights into vaccine design in the future.

The identification of additional sporozoite proteins that might be combined with PvCSP is of prime importance for a multi-component PE vaccine. *Plasmodium yoelii* sporozoite surface protein (PySSP3) is a newly characterized protein known to be essential for gliding motility [16]. Immunization of mice with PySSP2 and PyCSP is completely protective against the challenge with *P*. *yoelii* [51,52]. Here, anti-PvSSP3 seropositivity (43%) was detected during *P*. *vivax* infection and responses to this antigen were observed (37%) 360 days after infection. Anti-PvSSP3 antibody titers were stable in 30% (6/20) of subjects. Among tested PE antigens, PvSSP3 had the longest half-life (> 700 days). In addition, strong correlations were shown between antibody responses at day 0 to PvSSP3 and both PvCelTOS (Spearman ρ = 0.63) and PvM2-MAEBL (Spearman ρ = 0.70). At day 360, strong correlations between antibodies to PvSSP3 and both N- and C-terminal PvCSP (Spearman ρ = 0.60–0.63) were found. Together, our results indicate the immunogenicity of PvSSP3 to stimulate humoral immunity during infection. To address long-lasting anti-PvSSP3 antibody responses, further analysis on the stability of antibody responses and the generation of MBCs specific to this antigen in longitudinal studies are needed to support the half-life estimation. Possible explanations of correlation between anti-PvSSP3, anti-PvM2-MAEBL and anti-PvCelTOS antibodies that might require further investigation including: (i) the similarity of amino acid sequences that leads to stimulation of cross-reactive antibodies during infection; (ii) the timing of antigen release during parasite infection may be related. The PvSSP3-PvCSP correlation might indicate a co-localization of these antigens on the sporozoite surface with parallel induction of long-term antibody responses. Prior to inclusion of PvSSP3 in a PE vaccine, more studies of its molecular function and structural characteristics are required.

The sporozoite’s complexity and the expression of its various proteins contribute to its distinct biological functions. Here, sporozoite antigens which play roles in the traversal process (PvM2-MAEBL, PvSPECT1 and PvCelTOS) were assessed their antigenicity in vivax malaria patients. During acute infection, our results showed that the highest frequency of seropositivity was to PvCelTOS (70%), compared to PvM2-MAEBL (48%) and PvSPECT1 (30%). This was similar to findings in a previous study that showed naturally induced IgG responses to PvCelTOS in *P*. *vivax* patients [46]. Of these three antigens, anti-PvCelTOS antibody levels were higher than those specific for PvM2-MAEBL and PvSPECT1. In a year-long cohort study, only a few subjects maintained positive anti-PvCelTOS (10%) and anti-PvM2-MAEBL (30%) antibody titers, and none of the subjects had stable anti-PvSPECT1 responses. Antibody kinetics specific to PvCelTOS and PvM2-MAEBL demonstrated long half-lives (> 500 days). Together, our results indicated that while PvCelTOS has the ability to stimulate host immune responses in natural infection, the use of this antigen in PE vaccine design should consider the limited duration of its antibody response.

In the present study, assessment of IgG subclass responses to PvCSP-VK210 and PvSSP3 revealed that the major subclasses were cytophilic antibodies IgG1 and IgG3, with limited IgG2 and IgG4 responses. These cytophilic antibodies made up the preponderant antibody response, similar to previous studies using PvCSP and PvTRAP [45] and in *P*. *falciparum* asexual blood stage antigens [44]. Upon RTS/S vaccination, cytophilic IgG3 levels are associated with protection, followed by IgG1 levels [42]. A cohort study in Papua New Guinea (PNG) children revealed an acquisition of cytophilic IgG responses to *P*. *falciparum* reticulocyte binding-like homologue protein (PfRH5). The high IgG3 responders to this antigen had a stronger association with reduced risk of malaria during 1-year monitoring period [44]. From our result, the prevailing immune response involving cytophilic subclasses directed at sporozoite surface proteins, specifically PvCSP and PvSSP3, might serve a protective role against malaria through complement-mediated lysis [53] or cell-mediated mechanisms, such as opsonic phagocytosis and antibody-dependent cellular inhibition [54]. Accordingly, inclusion of PvCSP and PvSSP3 in PE vaccine design may induce the magnitude and persistence of IgG1 and IgG3 responses which effectively kill the sporozoites.

Here, we demonstrated the ability of *P*. *vivax* subjects to produce anti-PE antibodies against all tested PE antigens. Of 104 *P*. *vivax* acute subjects, 67 samples (64.4%) responded to at least three of PE antigens. Nine acutely infected patients (8.7%) produced antibodies against all tested PE antigens. In 1-year cohort study, 25% (5/20) of subjects showed seropositivity to five PE antigens and two PvCSP-VK210 subdomains during acute infection, whereas these subjects maintained seropositive responses to only PvCSP-VK210 for one-year period. Together, our study demonstrated an ability of symptomatic individuals to produce antibody responses against the surface, microneme and traversal protein of sporozoites following *P*. *vivax* natural infection. However, only antibodies to sporozoite surface proteins (PvCSP-VK210 and PvSSP3) could persist in most of our subjects for at least 1 year. Alongside with antibody response to PE antigens, individuals residing in endemic areas have been documented to exhibit elevated antibody responses to asexual blood stage antigens, including PvDBPII, PvMSP8 and PvRPB1a [55–57]. Therefore, the combination of both PE and asexual stage antigens in multistage vaccine development could be useful for inducing protective immunity against *P*. *vivax* infection.

It should be noted that our study has some limitations. A larger sample size with monitoring antibody levels in plasma within days or weeks after infection in the cohort study is required to confirm the kinetics and durability of total and subclass IgG responses to the tested PE antigens. Limitation also includes ascertainment of infection history of participants using records of Vector Borne Disease malaria clinics (Units 11.4.2 and 11.5.3), thereby missing those who had prior mild or asymptomatic infections. Therefore, our study could not demonstrate the relationships between individuals with *P*. *vivax* previous infection, as well as magnitude and durability of antibody responses. As repeated infections could boost long-term antibody and MBC responses, examining the dynamics of IgM and IgG antibodies over time and their correlation with memory B cell (MBC) function may provide valuable insights into the humoral immune response against the PE antigen. To gain better understanding of the humoral immunity against PE antigens following natural infection, it is required to establish the baseline of pre-existing IgG responses in individuals with prior *P*. *vivax* infections. This data is essential for evaluating the effects of boosting whether current infection could induce IgG levels and investigating correlation with ages, as it was well demonstrated in *P*. *vivax*-endemic PNG cohort study [58].

In summary, we demonstrated that natural antibody responses against PE antigens occurred and were persisted after acute vivax malaria. The seropositivity of PE antigens was greatest to full-length PvCSP-VK210 and PvCelTOS, followed by PvM2-MAEBL, PvSSP3, N- and C-termini of PvCSP-VK210, and PvSPECT1 (ranked by frequency of positive responders in cross-sectional study). Naturally-acquired antibody response to PvCSP-VK210 was the most stable seropositivity through one year. PvSSP3 had the longest half-life estimation. The pattern of IgG1 and IgG3 responses was predominant. In-depth study of immunogenicity of these two PE antigens in inducing long-lasting immune responses and their potential association with protection will be useful for the development of effective vivax malaria vaccine.

## Supporting information

S1 Table Characteristics of *P*. *vivax*- subjects and malaria naïve healthy donors recruited in this study. (DOCX)

S2 Table Details of PE antigens used for determination of serological responses in this study. (DOCX)

S1 Fig Experimental workflow and sample organization of this study. The presented study consists of a cross-sectional study and a 1-year cohort study. Dashed line refers to samples which were taken for further experiments. (a) Plasma samples of subjects who showed seropositive to PvCSP-VK210 and PvSSP3 were used for further assessment of IgG subclass responses. (b) Samples of subjects whose samples were available at four timepoints (Days 0, 90, 270 and 360) were taken for 1-year cohort study.
(TIF)

S2 Fig SDS-PAGE separation of purified recombinant pre-erythrocytic stage antigens used in this study. SDS-PAGE gel of (A) full-length PvCSP-VK210, (B) N-terminal PvCSP-VK210, (C) C-terminal PvCSP-VK210, (D) PvSSP3, (E) PvSPECT1 (F) PvCelTOS (G) PvM2-MAEBL.
(TIF)

S1 File Raw data for Figs 1–5. (XLSX)
