# Supplementary material for: Longitudinal analysis of antibody responses to Plasmodium vivax sporozoite antigens following natural infection
Source: PLoS Negl Trop Dis. 2024 Jan 26;18(1):e0011907. doi: 10.1371/journal.pntd.0011907 (PMC10817200; doi:10.1371/journal.pntd.0011907)
Supplement: S1 Table — (DOCX) [file pntd.0011907.s001.docx]

**S1 Table. Characteristics *of P. vivax*- subjects and malaria naïve healthy donors recruited in this study.**

| **Characteristics** | ***P. vivax* infected subjects** | | | | **Malaria-naïve, healthy subjects** |
| --- | --- | --- | --- | --- | --- |
|  | **at acute phase (day 0)** | **at recovery for  90 days** | **at recovery for  270 days** | **at recovery for  360 days** |  |
| **Total Number** | 104 | 41 | 30 | 27 | 52 |
| **Age (years)** | | | | |  |
| Median (Q1, Q3) | 29.5 (20.8, 42.0) | 28.0 (21.0, 39.0) | 32.5 (23.0, 42.3) | 30.0 (20.5, 43.0) | 26.5 (23.0, 30.3) |
| **Gender** | | | | |  |
| Male | 62% (64/104) | 63% (26/41) | 63% (19/30) | 59% (16/27) | 69% (36/52) |
| Female | 38% (40/104) | 37% (15/41) | 37% (11/30) | 41% (11/27) | 31% (16/52) |
| **Nationality** | | | | |  |
| Thai | 72% (75/104) | 78% (32/41) | 97% (29/30) | 100% (27/27) | 100% (52/52) |
| Myanmar | 28% (29/104) | 22% (9/41) | 3% (1/30) | 0% (0/27) | 0% (0/52) |
| **No. of prior infection** | | | | |  |
| 0 | 103 | 41 | 30 | 27 | 52 |
| 1 | 1 | 0 | 0 | 0 | 0 |
| **Parasitemia (parasite/µL)** | | | | |  |
| Mean ± SD  (Range) | 4759.98 ± 2160.79  (559.59 - 9533.68) | 0 | 0 | 0 | 0 |
