# Supplementary material for: Longitudinal analysis of antibody responses to Plasmodium vivax sporozoite antigens following natural infection
Source: PLoS Negl Trop Dis. 2024 Jan 26;18(1):e0011907. doi: 10.1371/journal.pntd.0011907 (PMC10817200; doi:10.1371/journal.pntd.0011907)
Supplement: S2 Table — (DOCX) [file pntd.0011907.s002.docx]

**S2 Table. Details of PE antigens used for determination of serological responses in this study.**

| **PlasmoDB accession no.** | **Abbreviated Name** | **Full Name** | **Location** | **Function in invasion** | **Important evidences related to infectivity inhibition** | **Reference** |
| --- | --- | --- | --- | --- | --- | --- |
| PVP01_0835600 | PvCSP-VK210 | *Plasmodium vivax* Circumsporozoite Protein-VK210 | Sporozoite Surface | host hepatocyte invasion | Monoclonal antibody to *P. falciparum* central repeat region of CSP showed inhibition of liver stage development | [15,20-23] |
| PVP01_1427900 | PvSSP3 | *Plasmodium vivax* Sporozoite Surface Protein 3 | Salivary gland and sporozoites surface | Gliding motility,  cell traversal and pre-erythrocytic stage development | - | [16] |
| PVP01_1212300 | PvSPECT1 | *Plasmodium vivax* Sporozoite Protein Essential for Cell Traversal 1 | Sporozoite microneme | Cell traversal | Individuals immunized by radiation-attenuated sporozoites confer anti-PfSPECT1 | [17, 18, 35, 37] |
| PVP01_1435400 | PvCelTOS | *Plasmodium vivax* Cell-traversal Protein for Ookinetes and Sporozoites | Sporozoite microneme | Cell traversal | *P. falciparum* CelTOS-immunized mice showed inhibition of sporozoite hepatocyte infection. PfCelTOS-specific monoclonal antibody inhibited oocyst development and transgenic parasites | [33, 34] |
| PVP01_0948400 | PvM2-MAEBL | *Plasmodium vivax* Membrane-associated erythrocyte binding-like protein | Sporozoite microneme, sporozoite surface and blood-stage merozoite rhoptry | attachment and invasion to mosquito salivary gland and host hepatocyte | PE development and lethal *P. yoelii* infection could be inhibited by anti-sera targeting the ectodomain M2 of MAEBL | [19, 29, 30] |
