# Supplementary figures and images for: Longitudinal analysis of antibody responses to Plasmodium vivax sporozoite antigens following natural infection
Source: PLoS Negl Trop Dis. 2024 Jan 26;18(1):e0011907. doi: 10.1371/journal.pntd.0011907 (PMC10817200; doi:10.1371/journal.pntd.0011907)

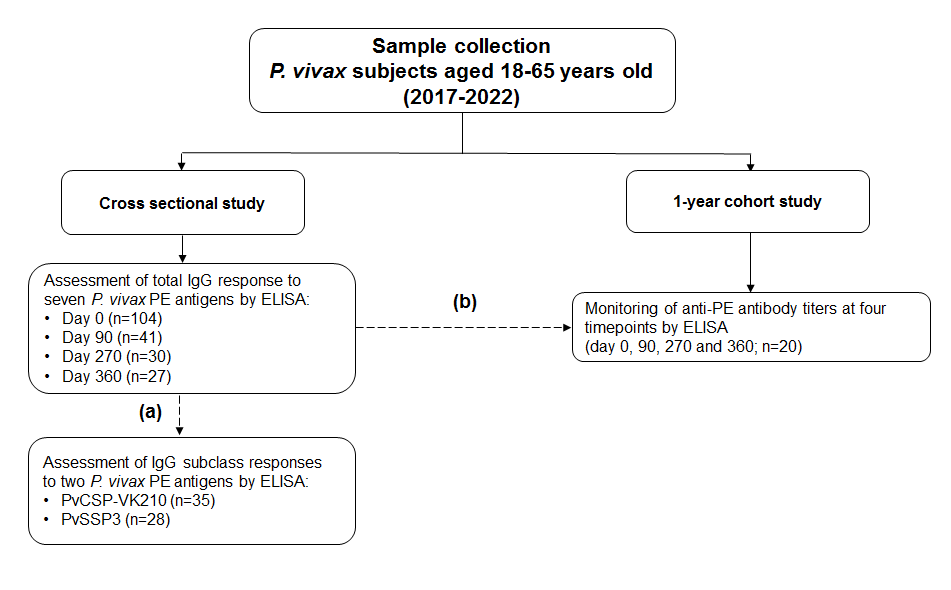

Supplement: S1 Fig — The presented study consists of a cross-sectional study and a 1-year cohort study. Dashed line refers to samples which were taken for further experiments. (a) Plasma samples of subjects who showed seropositive to PvCSP-VK210 and PvSSP3 were used for further assessment of IgG subclass responses. (b) Samples of subjects whose samples were available at four timepoints (Days 0, 90, 270 and 360) were taken for 1-year cohort study. (TIF) [file pntd.0011907.s003.tif]

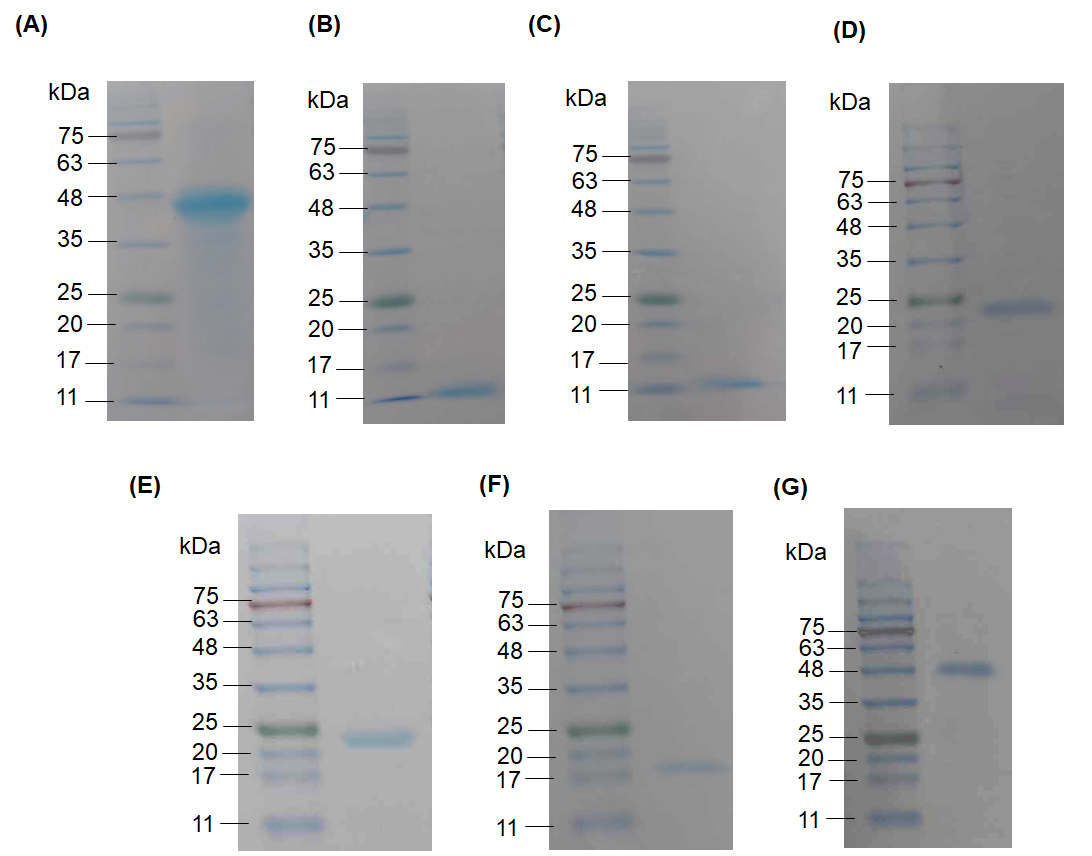

Supplement: S2 Fig — SDS-PAGE gel of (A) full-length PvCSP-VK210, (B) N-terminal PvCSP-VK210, (C) C-terminal PvCSP-VK210, (D) PvSSP3, (E) PvSPECT1 (F) PvCelTOS (G) PvM2-MAEBL. (TIF) [file pntd.0011907.s004.tif]
